# Adaptive Neuromorphic Circuit for Stereoscopic Disparity Using Ocular Dominance Map

**DOI:** 10.1155/2016/8751874

**Published:** 2016-05-03

**Authors:** Sheena Sharma, Priti Gupta, C. M. Markan

**Affiliations:** Dayalbagh Educational Institute, Dayalbagh, Agra 282005, India

## Abstract

Stereopsis or depth perception is a critical aspect of information processing in the brain and is computed from the positional shift or disparity between the images seen by the two eyes. Various algorithms and their hardware implementation that compute disparity in real time have been proposed; however, most of them compute disparity through complex mathematical calculations that are difficult to realize in hardware and are biologically unrealistic. The brain presumably uses simpler methods to extract depth information from the environment and hence newer methodologies that could perform stereopsis with brain like elegance need to be explored. This paper proposes an innovative aVLSI design that leverages the columnar organization of ocular dominance in the brain and uses time-staggered Winner Take All (ts-WTA) to adaptively create disparity tuned cells. Physiological findings support the presence of disparity cells in the visual cortex and show that these cells surface as a result of binocular stimulation received after birth. Therefore, creating in hardware cells that can learn different disparities with experience not only is novel but also is biologically more realistic. These disparity cells, when allowed to interact diffusively on a larger scale, can be used to adaptively create stable topological disparity maps in silicon.

## 1. Introduction

The ability to detect small differences in the interocular retinal disparities is critical for assessing the depth of objects and is crucial for survival in living beings. Even in artificial systems, the ability to perceive depth and distance are crucial for navigation, control, obstacle avoidance, depth measurement, environmental reconstruction, security, and so forth. While the precise biological mechanisms that compute depth from the relative position of the stimulus received by the two eyes is still largely unknown, different models have attempted to explain how 3D depth information could be extracted from the two-dimensional retinal projections. These models can be broadly classified into sparse or dense algorithms [1]. Sparse algorithms include methods that create sparse outputs. These algorithms employ explicit matching of different features such as segments, edges, and corners of the image seen by one eye with the other [2, 3]. The dense algorithms on the other hand produce dense outputs, they are area based, and they are classified as either local or global. The local methods are window based and compare the left and right images by defining a moving block or window of a definite size. Some examples of local methods are block matching based on Sum of Absolute Differences (SAD) [4], energy based techniques [5, 6], or phase based techniques [7]. Global methods operate on the image as a whole and are mostly energy based. They produce very accurate results; however, they take much longer computing time, for example, Dynamic Programming, Global Optimization, Intrinsic Curves, Graph Cuts, Nonlinear Diffusion, Belief Propagation, and Correspondenceless methods [8, 9]. Out of the sparse (feature based) and dense (area based) methods, while the feature based methods are more resilient to image variation, the area based methods are easier to implement, they can be interpolated, and the disparity can be calculated for every pixel in the image and therefore they are more widely used. Various hardware implementation of different algorithms that compute disparity has been proposed by various groups; for example, the authors in [10] use a digital approach using FPGA and IIR causal filters for phase based disparity estimation. However, they implement various hardware modules for performing tricky mathematical operations like multiplication, division, and squaring making their approach hardware intensive. Hariyama et al. [11, 12] propose a digital approach that uses the SAD method. They employ Laplacian of Gaussian filters to create a system with an adaptive window for stereo correspondence to increase disparity estimation quality. Another digital approach proposed by [13] uses a modified phase based technique to create a SOC using FPGAs that can be used in embedded systems. While digital approaches are known for their accuracy and speed, it has been emphasized that analog approaches more closely replicate the computations in the brain [14] and are ideal when it comes to emulating local computations in the brain [15]. Some purely analog models based on sparse disparity computations are there in literature, for example, [16, 17]. These models use WTAs and comparator circuits to estimate disparity. However, although analog, these models do not seem to take any inspiration from the structure or functioning of the brain.

Another category of hardware models (not purely analog) that derive inspiration from some aspect of neural computations also exists; for example, [18–20] propose mixed analog digital hardware based on the binocular energy model that takes inspiration from the hierarchical organization of the visual cortex to develop disparity tuned complex cells from simple cells. Another interesting hardware/software codesign for disparity computation that emulates the asynchronous event based, sparse coding in the brain can be found in [21]. But even though this implementation is bioinspired in some way, it does not take true advantage of the structural and functional elegance with which the brain is designed. Therefore, there is a need to look at newer approaches that take advantage of both structural and functional organization in the brain and only then can we make true progress in neuromorphic design.

*(1) Need for Adaptable Neuromorphic Stereoscopic Algorithm.* Nature has devised amazing ways to reduce wiring length, processing time, and power consumption by ensuring that most of the computations are carried out locally by means of a few neurons organized in a hierarchical fashion with minimum long range connections. For this the brain uses topographic mapping. This topographic mapping is present in all sensory systems. In the visual system topographic mapping ensures that adjacent spots on the retina are represented by adjacent neurons in the lateral geniculate nucleus and the primary visual cortex. One outstanding example of this is the ocular dominance map observed in the visual cortex. This topographic organization ensures that the neurons corresponding to the same spatial location in the left visual field and the right visual field are mapped close to each other on the cortex. It is conjectured that the functional significance of ocular dominance patterns is in 3D vision or the perception of depth. This is supported by experiments that have shown that abnormally reared animals, with only one functional eye, do not ever develop the ability to perceive depth [22]. Therefore taking advantage of ocular dominance (OD) to compute disparity information would imply looking at disparity the way the brain does. An algorithm that computes disparity on the basis of OD has been proposed by [23]; however, this method is difficult to implement in hardware because it uses complex computations involving Fourier transforms and logarithms to compute disparity. It is most unlikely that the brain would use such complex computations to extract disparity and therefore there is a need to look at more biologically realistic approaches.

Recent experimental evidence suggests that the perception of depth comes in as the infant is exposed to its environment and is not present at birth [24–27]. Therefore it seems that like ocular dominance and orientation selectivity, disparity selective neurons also tune their responses over a period of time after receiving inputs from the two eyes. Based on this adaptive mechanism a new class of neural network based models has emerged. These models develop disparity selective cells or filters from experience and map their responses to disparity outputs and hence exhibit flexibility and adaptability to work in different environments [28–31]. These approaches do not match the left and right images; instead, disparity is detected (by means of heightened response) by these trained disparity selective neurons when binocular stimuli with a specific disparity are fed into them. Therefore these models represent a class of adaptive algorithms that take inspiration from cortical plasticity. However, there has been no hardware implementation of these models.

In this context, the work presented in this paper explores a novel approach, using purely analog hardware, to build a disparity selective neuron, which is closer to biology, since it leverages the organization of ocular dominance columns to create an adaptive cell based on time-staggered Winner Take All competition implemented using floating gate pMOS dynamics [32]. Floating gate based analog hardware emulates synaptic dynamics very closely and has been used in various neuromorphic applications for introducing adaptation [33] and long-term memory [15, 32, 34]. It has also been used by us to create adaptive feature maps for ocular dominance and orientation selectivity [15, 32]. In this paper, for the first time, we move one layer up in the cortical hierarchy to build cells that take inputs from the ocular dominance patterns and tune their response to different disparities. This work is novel because it is the very first attempt to create disparity tuned cells in analog hardware which are adaptive; that is, they can learn from experience and are truly bioinspired as they take advantage of the hierarchical and layered architecture of the visual cortex.

The simulations were performed using Tanner T-Spice v13.0 and Cadence Specter v7.1 with BSIM3 level 49 spice models for 0.35 *μ*m CMOS process. The authors recommend that the reader should also refer to [15, 32] for complete understanding and appreciation of the work presented here.

## 2. Neural Development, Synaptic Competition, and Time-Staggered Winner Take All

Synapse pruning is a well-accepted mechanism underlying mammalian neurological development. In infants there is huge excess of synaptic connections but these synapses lack strength and precision. As development progresses, some synapses strengthen and mature and some are removed like removing weak branches to strengthen a tree. Because of this pruning and refining of neural connections, the 1000 trillion or so synapses present in young children are trimmed to about 100 trillion to 500 trillion by adulthood. Whether a synapse is maintained or not is determined by the level of activity in the synapse. During the prenatal period synaptic activity comes from the spontaneously generated nerve impulses, whereas after birth the synaptic activity is primarily due to the sensory input from the environment. Such inputs include visual stimulation, sound, and touch, which activate neurons to fire impulses that converge on a postsynaptic cell. Synapses between neurons that work together are strengthened, whereas synapses between cells that are not synchronized are eliminated [35–39]. To be more precise, when activity at two synapses is separated by 20 ms or less, the activity is perceived as synchronous and the elimination is prevented [40]. While synapse pruning occurs throughout our lifespan when we are subjected to new stimuli or we acquire new skills, majority of this synapse refinement occurs during a window of opportunity called the critical learning period during early development [41]. This critical period varies for different regions of the developing brain and during this period specific neural centers are especially receptive to incoming stimulation. In the presence of appropriate stimuli, these centers flourish by strengthening and fine tuning their synaptic connections. Therefore strengthening of synapses that are active and elimination of synapses that are inactive are the hallmark of neural development. This phenomenon of synapse elimination as a means of honing neural connections is also appropriate for purely analog VLSI implementation because while it is possible to stop using some connections, it is not possible to create new ones dynamically in hardware.

Synaptic competition occurs since the total synaptic area that a neuron can support is metabolically constrained. Therefore, when synapses from many different neurons are innervated by a postsynaptic cell, these synapses compete for resources. The synapses that are more active take up these resources and the inactive synapses that do not draw any resources get more and more weakened and ultimately reach a stage of no recovery or elimination. Usually a single neuron makes not just one but many synaptic connections with a postsynaptic neuron. Therefore when many presynaptic neurons have their synaptic arbors connected to a postsynaptic cell, it is only through uncorrelated activity between the several presynaptic cells that the postsynaptic neuron can tell from which presynaptic neuron the activity is more. Therefore if activity of all presynaptic neurons and the postsynaptic neurons is correlated, synaptic competition is prevented; however, when the activity is uncorrelated, competition is enhanced [42]. This uncorrelated activity between synapses belonging to neurons that respond to different features of the stimuli is also vital for feature map formation as has been argued by us previously [15, 32].

**Figure 1:**
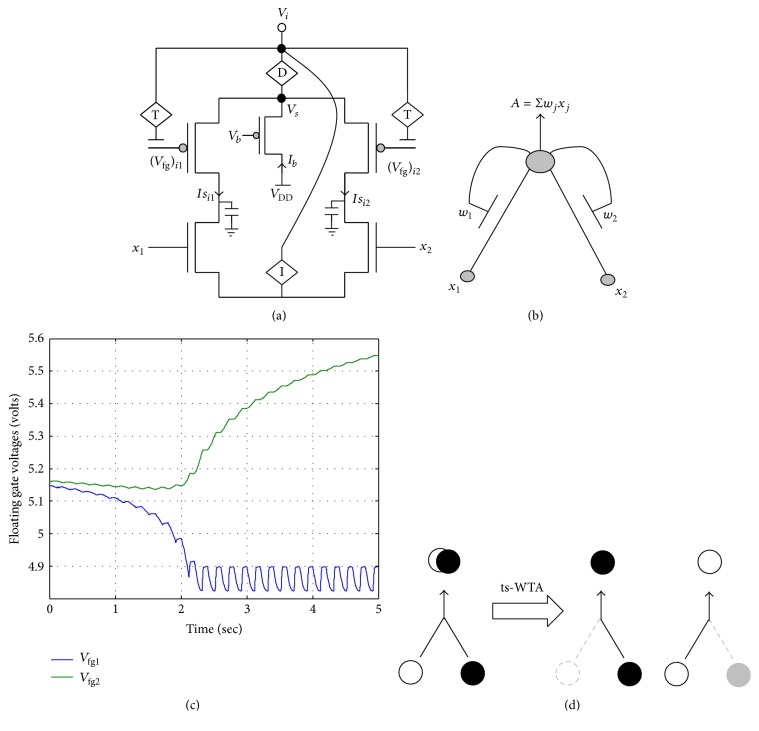
(a) Actual circuit of the ts-WTA learning cell and (b) its abstract model. In (a) (*V*_fg_)_*i*1_ and (*V*_fg_)_*i*2_ and in (b) *W*_1_ and *W*_2_ show the floating gate based weighted connections. *x*_1_ and *x*_2_ are inputs and node voltage *V*_*i*_ is activation of the cell which is equivalent to *A* in (b). (c) shows ts-WTA evolution of floating gate voltages. (d) Starting with nearly equal weak connections (left), the cell strengthens the stronger of the two connections at the cost of the other (right, shows both possibilities). Here ○ implies connection representing one feature and ● implies connection representing other features (adapted from Gupta and Markan, 2014, [15]).

Based on these fundamental concepts underlying neural development, that is, resource limitation, synaptic competition, uncorrelated activity, and synapse elimination, a truly bioinspired and novel analog CMOS design of a time-staggered Winner Take All circuit (Figure 1) has been proposed in [32]. This circuit, which is built on the adaptation dynamics of floating gate PMOS synapses, performs “time-staggered” (spread over time) competition between two arms that represent synapses bound to different neurons that connect at a postsynaptic neuron which has limited resources. When stimulated alternately or in an uncorrelated manner, these synapses compete for the limited resource (fixed amount of current, *I*_*b*_, through the bias pFET). If both the synapses are stimulated equally, the synapse with a stronger bias (lower initial floating gate voltage) wins; however, if the stimulation is unequal, the synapse that is stimulated more emerges as the winner. The synaptic weight, which is the floating gate voltage, is changed by two antagonistic quantum mechanical processes of injection and tunneling. Injection decreases the floating gate voltage by injecting electrons on it, while tunneling removes electrons from the floating gate thereby increasing the floating gate voltage. If during the overall learning phase tunneling is more than injection, the synapse gets eliminated, and if injection is more than tunneling, the synapse emerges as the winner. To ensure that the floating gate voltages or synaptic weights change according to the level of activity of the synapses, feedback devices 〈T〉 and 〈I〉 (in Figure 1(a)) have been devised. The circuit description and the details of their operation can be found in [32]. A detailed mathematical analysis of the dynamics of ts-WTA can be found in [32] and for a short description of the salient features of ts-WTA and its comparison with other WTA circuits please refer to section 2 of [15]. Here we reiterate some of the prominent features of the ts-WTA which make it unique. The ts-WTA can perform competition between inputs that are uncorrelated or not applied at the same time. In all other WTA circuits [33, 43, 44] the competition can happen only between inputs that are applied at the same time. Since uncorrelated inputs are essential for feature map formation [15, 32] only ts-WTA can accomplish brain like feature map formation. Due to the long-term charge retention capability of floating gate MOSFETs, ts-TWA has a memory element unlike other WTAs and therefore is ideal for hardware implementation of long-term memory.

This ts-WTA competition can be extended to any two opposing input synapses, for example, left/right eye connections in ocular dominance, ON/OFF cells in orientation selectivity, and Lagged/Nonlagged cells in direction selectivity, and could also be extended to other sensory modalities. By embedding these ts-WTA cells in an RC grid, we have been able to achieve diffusive interaction and cluster formation. An application of ts-WTA in forming ocular dominance maps can be found in [32] and another application of ts-WTA in forming orientation selective cells can be found in [15] and we propose that this ts-WTA competitive cell truly emulates brain like computing and can be used as a basic building block for recreating artificial feature maps in silicon of various sensory modalities as seen in the brain. In this paper, for the first time, we use ts-WTA to model a hierarchically superior layer of neurons that take information from the first layer of the cortex, that is, the ocular dominance pattern, and learn to detect different disparities that facilitate 3D depth perception. The next section discusses the design and working and salient features of the disparity selective cell developed.

## 3. Proposed Disparity-Learning Algorithm

### 3.1. Disparity-Learning in the Brain

While the anatomy of the visual system appears to be only two-dimensional, somewhere in the nervous system information of the third dimension is extracted from the retinal projections formed by the left and the right eyes as a result of their viewing the world from slightly different directions [46]. It is now well accepted that the brain computes the relative depth of objects based on the disparity in the relative horizontal position of the objects in the two eyes. It has also been shown that binocular disparity is the sufficient cue for stereoscopic depth computation [47, 48]. Further, physiological experiments reveal that a substantial number of neurons in the cortex detect horizontal positional disparities of retinal images [49, 50]. These disparity-sensitive neurons have been found in all extrastriate cortical visual areas of the macaque, from V2 to V5, and in even higher proportion than in V1 [49–53]. Additionally, some behavioral experiments suggest that new born humans and monkeys are unable to detect objects in random stereograms. It seems that in monkeys stereopsis appears to emerge after 4 weeks [24] and in human babies it appears after about 4 months [25, 26]. Therefore, it has been hypothesized that before these ages the inability to perceive depth is attributable to the absence of disparity tuned neurons in V1 [27]. Therefore, it seems that disparity tuned neurons emerge as an outcome of stabilization of neural circuitry between the two eyes and the cortex on repeated stimulation of the two eyes over a period of time after birth and therefore it can be said that during early development certain neurons learn different disparities on binocular stimulation. Hubel and Wiesel proposed a hierarchical model of cortex, wherein information is processed in a bottom up fashion from simple to complex cells. The early layers of the cortex extract basic features and subsequent layers use these basic features to process more complex features. On similar lines, disparity detection could be considered to be a multistep process in which the first layer extracts the left and right eye image properties and the next layer estimates the disparity and an even higher layer computes 3D depth. Additionally, during early development or critical learning period, the neurons tune themselves to different disparities they are exposed to and later respond to those disparities present in the visual stimulus (Figure 3).

Many models for estimating the disparity have been discussed in Section 1, but as pointed out none of them are truly bioinspired in the context of taking inspiration from the architecture of the brain. However, a model for binocular stereo segmentation that captures to some extent the essence of columnar architecture of the mammalian brain called the cepstral model is reported in [23]. This model takes advantage of the columnar interlacing of the cortex to develop a purely parallel algorithm for real-time stereo segmentation. Our model is inspired by the cepstral model in taking advantage of the ocular dominance columnar architecture; however, we distance ourselves from cepstral model by employing an adaptive hardware for disparity learning. The algorithm in [23] on the other hand is not adaptive and is difficult to implement in hardware because it uses complex mathematical functions such as logarithms and Fourier transforms.

### 3.2. Proposed Design of Adaptive Disparity Selective Cell

**Figure 2:**
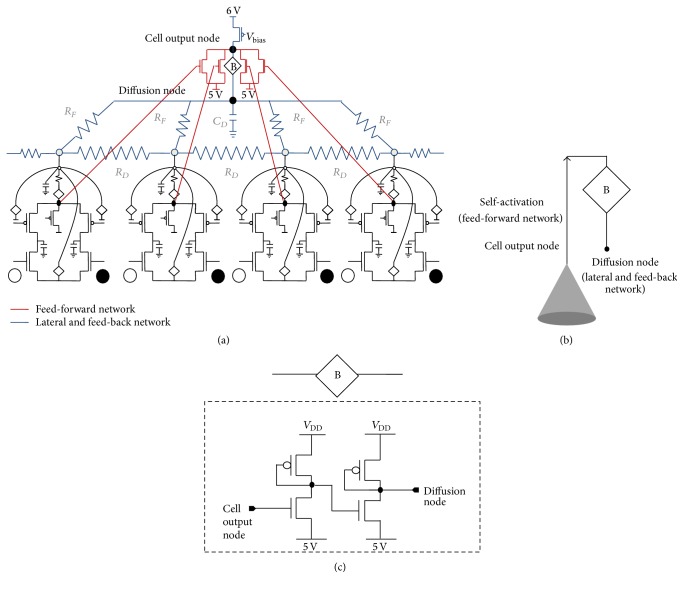
(a) shows a 1 × 4 subsection of the 9 × 9 receptive field of ts-WTA cells that form a disparity selective cell. The output of each ts-WTA is connected in a feed-forward manner using MOSFETs with their drains connected together giving out the response of the cell. The output of each cell is also connected at the diffusion node with feedback resistances (1 k each). The cell's output and diffusion nodes are separated by a buffer device 〈B〉 that only allows the cells output to affect the diffusion node voltage but the diffusion node voltage cannot affect the output node voltage directly. The bias transistor acts like a current source with fixed resources. (b) shows the symbol for the disparity selective cell. (c) shows the circuit level description of the 〈B〉 buffer device (adapted from Gupta and Markan, 2014, [15]).

**Figure 3:**
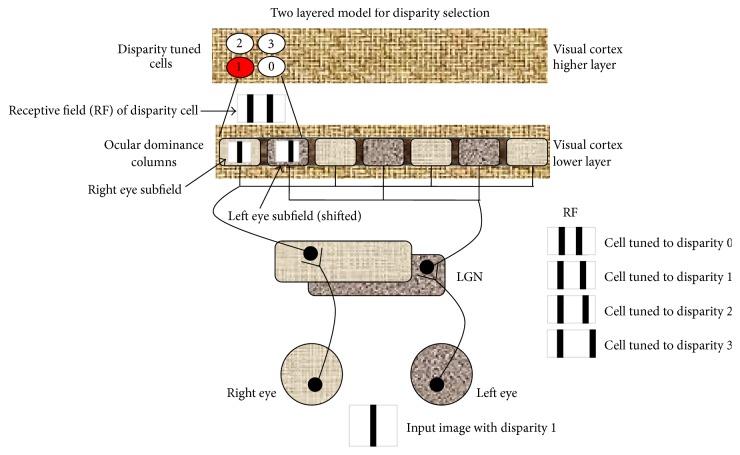
A 2-layer hierarchical model of disparity tuning and selection. When an image is shown to the two eyes, depending on its disparity, its projection in one of the eyes is shifted. The receptive field of the disparity tuned cell is composed of subfields from the two eyes projected on the lower cortical layer. Therefore, for different disparities and different shifted subfields, different disparity tuned cells respond. Both the layers of the cortex are topographically arranged.

The architecture of the disparity selective cell is similar to the orientation selective cell described in [15]. The receptive field of a disparity cell is composed of 9 × 9 ts-WTA cells. A 1 × 4 subportion of the 9 × 9 receptive field is shown in Figure 2. The output of each ts-WTA is connected through MOSFETs whose drains connect at a common node which is the cell's output node. This is the feed-forward network of the cell. A buffer device 〈B〉 connects the output node of the cell to the diffusion node. This buffer device conveys the voltage at the cells output node to the diffusion node; however, it does not allow the voltage at the diffusion node to affect the cell's output directly. This becomes important when many such cells are diffusively connected with each other. All the resistances *R*_*D*_ and *R*_*F*_ are 1 kohm and the capacitor *C*_*D*_ is 10 pF. The resistances *R*_*D*_ diffusively couple the output of each ts-WTA with its neighbors so that local clusters are formed and the resistances *R*_*F*_ feed the output of the disparity cell back to the individual ts-WTAs so that the input patterns for which the response of the cell is high can get correctly reinforced on the individual floating gates through the feedback mechanism of each ts-WTA cell.

While the design and functioning of the disparity cell are similar to the orientation cell described in [15] and both the cells learn one of the input patterns applied to them depending on their initial biases, one major difference is that the disparity cell is at a hierarchically superior position and the input it receives is from the first layer of cortex (and not the retina as in [15]). The input in this case is the receptive field of disparity cell, which is in the form of an interlaced pattern, wherein half part is from the subfield belonging to the left eye and the other half is from the right eye (extracted from the ocular dominance pattern). In the beginning of the simulation, the receptive field of disparity cell [i.e., 9 × 9] is given random initial biases within 4.8 V–5.5 V. A set of input patterns resembling 4 different disparities were created, as the 9 × 9 receptive field can accommodate only 4 different disparities (0, 1, 2, and 3). Each pattern from the set is comprised of 9 × 9-interlaced image, where the bright (ON) part of the image represents a high voltage (+6 V) pulse and the dark (OFF) portion represents a low voltage (−1 V) pulse with a pulse width of 0.02 s. To make sure that the leaning is not biased towards any particular disparity, generated input patterns from the set are applied to the receptive field of disparity cell iteratively in a random-inside-epoch [15, 32] manner. During this iterative process, it has been ensured that in each ts-WTA the two opposing synapses are stimulated alternately. This is made possible by stimulating the two synapses by complementary patterns [15], so that when one pFET synapse is ON (gate voltage −1 V) the other is OFF (gate voltage 6 V) and vice versa. This leads to ts-WTA competition between the individual ON/OFF synapses and one of the disparities gets selected in a way similar to orientation selectivity shown in [15]. Depending on the initial bias of the cell, each pattern evokes a certain response in the cell in the form of an output voltage. This output voltage is fed back to the individual ts-WTAs and the feedback regime of each ts-WTA cell modifies the floating gate voltage of each synapse appropriately. The input pattern that evokes the maximum response is the pattern that the cell eventually learns.

**Figure 4:**
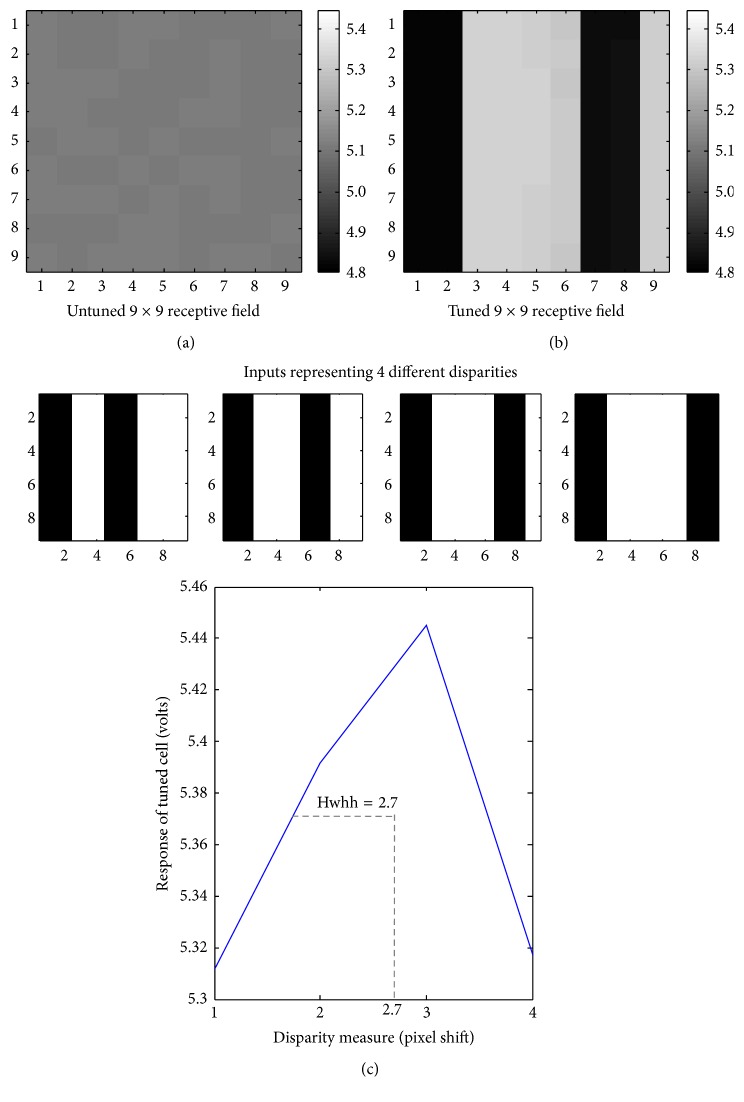
(a) represents an undeveloped receptive field with random initial biases. (b) represents the developed floating gate voltages or the disparity tuned receptive field after the learning phase is over. (c) shows the tuning curve for the disparity selective cell. Out of all the disparities it has the maximum response for disparity 3; therefore we say it is tuned for disparity 3. It is tuned at hwhh = 2.7 which is a good tuning measure.

Therefore, total eight different input patterns (4 disparity patterns and 4 of their complementary patterns) in a random epoch manner are applied to the disparity cell for 80 epochs and as the simulation progresses the disparity cell learns one of the disparities from given set. The cell works in two phases: (i) the disparity-learning phase (emulating the critical learning period in the developing cortex) and (ii) the disparity-detecting phase (emulating the adult cortex). In the learning phase, inputs patterns with four different disparities (disparities 0, 1, 2, and 3) and their complementary patterns are used to stimulate the disparity selective cell. The learning phase typically takes 3 to 4 seconds. Once the disparity is learnt, the cell acts like a disparity detector giving high response whenever an image pattern with the same disparity is shown to it. The cell takes around 0.001 s to detect the disparity, qualifying as a real-time disparity detector.  Figure 4 shows how starting from a randomly biased 9 × 9 receptive field the disparity cell develops a receptive field of disparity 3. As the receptive field of disparity cell develops, there is an increase in response for a particular disparity, which can be seen from the sharpness of the tuning curve.  Figure 4(c) shows that the response tuning (maximum response) of the cell is at disparity 3. Different cells can be tuned to different disparities and hence we could have an array of cells tuned to different disparities for each small segment of the image to ensure retinotopic mapping and local computations (Figure 4(c), top).

### 3.3. Experiments and Results

**Figure 5:**
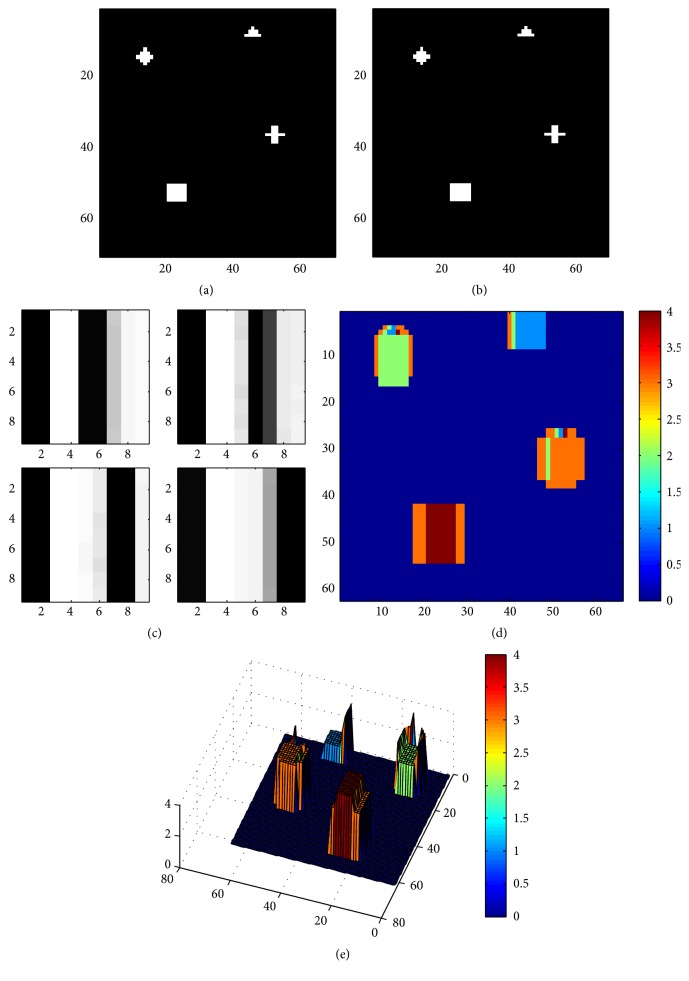
(a) and (b) Synthetic left and right stereo images with 4 disparities. (c) shows the 9 × 9 receptive fields of 4 disparity cells tuned to different disparities that are used as filters to extract disparity from (a) and (b). (d) represents the obtained disparity map. (e) represents 3D reconstruction of the disparity map showing the exact depth of each subfigure.

The perception of depth in stereo image depends on the correct matching of corresponding patches between left and right images. The match is along the epipolar line because the interest is only in the horizontal disparities. This would also help in reducing the ambiguity. The disparity selective cells tuned to 4 different disparities (Figure 5(c)) can now be used to detect the disparity in a stereo image which is comprised of 4 different disparities. Figures 5(a) and 5(b) are set of stereo pair images of size 80 × 80. In order to find the disparity of every pixel using disparity selective cells, the patch of size, “height (*h*) × width (*w*),” is extracted from both left image and its corresponding right image. Extracted patches from stereo images are then joined together along one edge to form a window of size *h* × 2*w*. This resembles the pattern found in layer IV of primate visual cortex, in form of ocular dominance columnar pair. In the present case, the size of spliced image should be 9 × 9, which means that size of 9 × 4 from left image is spliced along the size of 9 × 5 from right image. The disparity selective cells are applied on each such window which consists of portion extracted from left image and the right image with the columnar width as maximum disparity. Out of four disparity selective cells, one with the same disparity will have the highest response and is the winner. The disparity corresponding to the winner cell would be the disparity of that pixel. Figures 5(d) and 5(e) represent the obtained disparity map and its 3D reconstruction, respectively.

**Figure 6:**
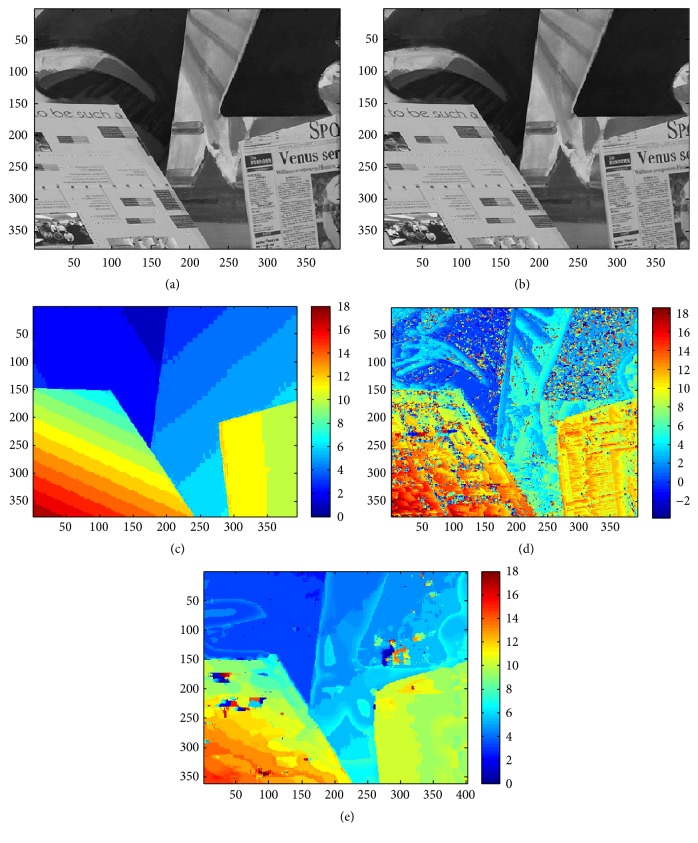
(a) and (b) represent left and right stereo images with 20 disparities. (c) represents the true disparity map. (d) represents the obtained disparity map by using disparity cells tuned to 20 different disparities as filters; average error is 68%. (e) represents the improved map obtained by adding edge detection filters; average error is 22%.

As the receptive field size increases, the number of disparities that can be represented also increases. To illustrate, 20 receptive fields of disparity cells, each of size 10 × 40, were developed, which corresponded to the 20 disparities, in a similar fashion. Now total 40 different input patterns (20 patterns corresponding to the disparity and its 20 complementary patterns) are applied to the disparity cell (with random initial values between 4.8 V and 5.5 V) in a random epoch manner for 80 epochs. As the simulation progresses, the disparity cell would learn one disparity out of 20 disparities from the given set. Once the disparity is learnt, we get 20 such disparity selective cells that can be used to find disparities from 0 to 19. We have applied these 20 disparity selective cells on a benchmark stereo image, which has maximum disparity as 20. In this case, 20 disparity selective cells are applied on each such spliced image of size 10 × 40 (10 × 20 from left and right image each). To reduce the noise, the spliced pattern of size 10 × 40 is applied to the Gaussian filter whose output is more stable with respect to noise in the stereo image. Figures 6(a) and 6(b) show the left and right stereo images with 20 disparities, Figure 6(c) shows the true disparity map, and Figure 6(d) shows the obtained disparity map.

While this resolution is only for low level vision, this can be improved by additional filtering mechanisms like edge detection filtering. This method helps to identify the discontinuity that corresponds to abrupt changes in the image. The stereo image is first filtered through edge-detector filter and then the disparity selective cells are applied to the spliced image. This filtering would improve the result in terms of disparity map of the stereo image as shown in Figure 6(e). The field size is increased in accordance with the increase in the number of disparity levels intended to detect. A 10 × 40 field size will be suitable for any image size given that the disparity levels in the image are in the range from 0 to 19.

### 3.4. Comparison

Over past few decades, conscious efforts have been made by researchers to study the field of stereo vision with the aim of gaining greater insight into visual perceptual mechanisms that nature has optimized. These models explain how 3D depth information could be extracted from the two-dimensional retinal projections and can be broadly classified into sparse or dense algorithms [1].

The major impediment in working with these computer based software approaches is the serial behavior, which has limitations in real-time processing. To overcome this limitation, the recent stereoscopic research uses dedicated hardware platform for real-time stereo vision, such as digital signal processors (DSP) [10–12], field programmable gate arrays (FPGA) [13], and application-specific integrated circuits (ASIC) [16, 17]. The implementation of these offline stereo algorithms in hardware board results in better efficiency as compared to serial software stereo vision algorithms.

Regardless of these astounding advances to match the human visual processing, one continues to be humbled by salient features of the brain. These offline computational approaches and hardware are not enough to understand the way that several different tasks are performed in the visual cortex. There are many models, which either try to depict the functioning of brain or design the model on the principle based on biological way of extracting disparity. Like receptive field base algorithms, which are used to detect the visual information, for instance, edges and features can be utilized in an integrated visual system [18–20]. All these algorithms suffer from inability to adapt to experience. None of these approaches takes genuine benefit of the layered architecture and information organization in the brain and none of them exactly imitates the developmental or adaptation aspects of the brain.

The solution lies in learning based algorithms that mature the filters that amalgamate other visual information and adjust themselves to the changes in surroundings. There are models representing a class of adaptive algorithms that take inspiration from cortical plasticity; however, there has not been any hardware implementation of these models [2, 31, 54]. In this paper, we propose an analog design for adaptive disparity selective cells; that is, they can learn from experience and be used to detect disparity in stereo images. This approach is truly bioinspired as it takes advantage of the hierarchical and layered architecture of the visual cortex.  Table 1 summarizes the comparison of the various hardware based disparity algorithms with the proposed method.

In this paper, we opt to give less weightage to efficiency as compared to adaptability, robustness, and self-learning. To the best of our knowledge, no other model takes inspiration from both the structure and function of the brain and no other model shows cortical plasticity or adaptability the way our implementation does. Although the resolution is only for low level vision right now, this can be improved by adding additional filtering mechanisms. The training phase typically takes 3-4 seconds, while the detection phase takes around 0.001 s for any pattern, hence suitable for real-time implementation. The proposed design also has lower power dissipation than the other hardware models (data for which is available).

This work introduces a novel concept of disparity-learning cells, which is purely original and has never been developed in analog hardware ever before. The approach has biological propinquity, since it captures the essence of both the developing and the developed human brain.

## 4. Diffusive Interaction of Disparity Cells

Evolution ensured that the brain optimizes on power consumption and processing speed while remaining small in size by ensuring that most of the computations happen locally so that wire lengths could be minimized. This was achieved through topological mapping and formation of feature detectors that are smoothly spread over the whole cortical structure. Various topological maps (patterns of synaptic connections), like ocular dominance, orientation selectivity, direction selectivity, and so forth, have been reported in the visual cortex [55, 56]. All these feature maps have three characteristics in common, continuity, diversity, and global order. Continuity means that nearby neurons show similar feature preference that varies smoothly across the cortical surface. Diversity implies that there is equal representation of all features over the cortical surface and global order ensures there is periodic organization of all features on the cortical surface. In a similar way, disparity maps have also been observed in the visual cortex [45]. These maps are critical to the functioning of the brain and most of them are formed during a critical learning period [41]. If for some reason the organism receives abnormal inputs during this critical learning period, there is malformation in the cortical feature maps and that function is impaired [22].

**Figure 7:**
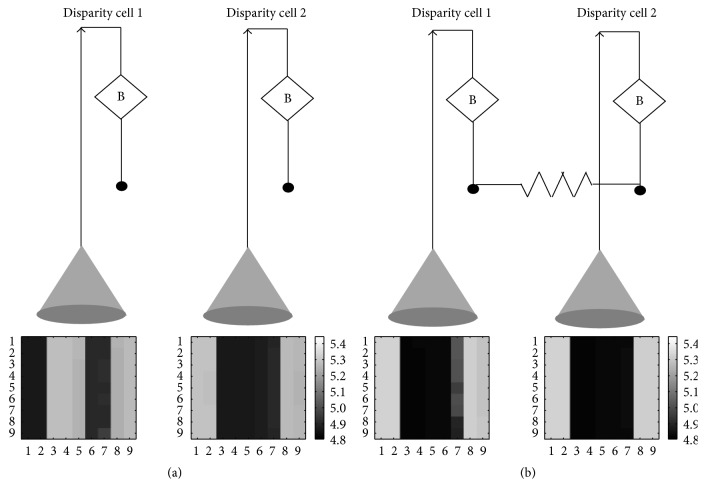
(a) represents two disparity cells that are allowed to develop under individual bias but no neighborhood influence. They usually develop to have different disparity tuning. (b) When the same disparity cells are connected by means of a diffusive resistor (100 ohms), the more strongly biased cell influences the development of the other cell and they form cluster of the same feature preference. On a larger scale this leads to the formation of feature maps.

In this section, we will discuss the ability of the disparity selective neurons to form clusters, which makes them suitable for map formation. In our previous papers [15, 32] we have used Diffusive-Hebbian learning, which is based on the biological phenomenon of Reaction-Diffusion to model ocular dominance and orientation selectivity maps. Here, we attempt to apply the Reaction-Diffusion framework for formation of disparity maps with disparity selector cells. The essence of working is that if the disparity selective cells have overlapping receptive fields and they receive similar inputs, then they are forced to have similar responses. Hebbian learning will confirm that these cells form a cluster. In the brain, it is conjectured that diffusion acts by means of leaking chemicals from an active cell which reduce the threshold of neighboring cells making them fire more readily and thereby making clusters of nearby cells that fire together. In hardware, Reaction-Diffusion can be implemented by means of an RC network and we have used it successfully for ocular dominance and orientation selectivity [15, 32]. Similar RC network has been used in this paper to connect two disparity selective cells.  Figure 7 shows the development of two cells with and without diffusive interaction. As can be seen, when the cells are not connected they develop to have preference for different disparities; however, when they are connected at their diffusion node, the cell with the stronger bias influences the development of the other cell and they both learn to respond to the same disparity. Fundamentally, the disparity selective cell learns one out of the four different disparities that it is stimulated with during the learning phase according to its initial (genetic) biases.

## 5. Disparity Map Formation

The above-mentioned diffusion process between the disparity cells is responsible for the formation of clusters and can crudely be modeled by means of a polynomial whose stable roots represent different disparities:

(1) X=x−disparity+1⋯x⋯x+disparity+1.

**Figure 8:**
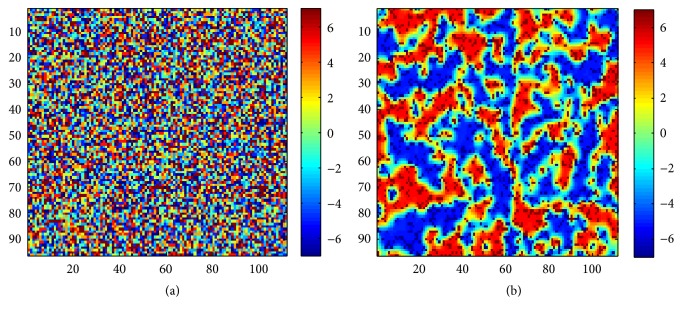
(a) Development of disparity tuned cells, starting from random initial biases for disparities, in the absence of diffusive interaction with neighborhood. All cells develop under the influence of individual initial biases only. (b) The same cells when allowed to develop under diffusive neighborhood influence grow in clusters of cells with the same disparity preference with disparity tuning varying smoothly across the clusters, therefore demonstrating* continuity*,* diversity*, and* global order*.

In the above given equation, for different values of *x*, the equation stabilizes to different roots analogous to learning different disparities. Therefore, if we take a patch of the cortex, where each cell is a disparity-learning cell, and allow each cell to develop according to (2), then according to its initial bias each cell falls into one stable root representing its disparity (Figure 8(a)):

(2) $$\begin{array}{c} X_{new}=X+\alpha \left(\;X\;\right), \end{array}$$

where *α* is reaction constant.

However, when a diffusion term is added (see (3)), neighborhood influence starts to act on each cell and clusters of cells of the same disparity start emerging. Once the learning period is over, periodically distributed clusters representing different disparities can be seen. Between different clusters there is gradual variation in the feature preference:

(3) $$\begin{array}{c} X_{new}=X+\alpha \left(\;X\;\right)+D_{u}\left(\;X\;\right), \end{array}$$

where *α* is reaction constant and *D*_*u*_ is diffusion constant.

**Figure 9:**
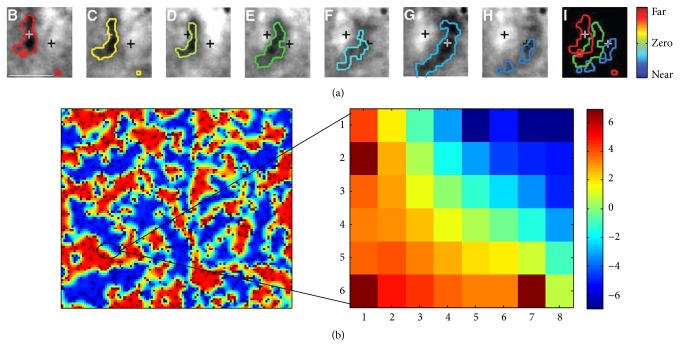
(a) Biological disparity map of neurons in cortex of macaque monkeys evoked by stimuli with seven different disparities. The position of the two crosses is constant through all the images marked as (B)–(H) (adapted from [45]). (b) A small portion extracted from the obtained disparity map to show consistency with the recent physiological discoveries about the smooth change of stimuli preference (*continuity*) in topographic maps in the brain as shown in (a).

Here, the map created by the disparity selective cells with diffusive interaction exhibits continuity, diversity, and global order, thus fulfilling the three important tenets of the feature map in the brain. Further, a small portion extracted from the obtained disparity map of Figure 8(b) as shown in Figure 9(b) shows the consistency with the recent physiological discoveries about the smooth change of stimuli preference (*continuity*) in biologically observed disparity maps [45]. Therefore, the Reaction-Diffusion framework with the proposed adaptive disparity selective cells is effective in forming clusters and hence is suitable for the formation of disparity maps found in the brain.

Recreating these maps adaptively in silicon has huge potential in areas such as robotics, artificial vision systems, and even cortical prosthesis, where damaged portion of the cortex could be replaced by adaptive silicon chips that could fine-tune to the specific environment and perform the same function.

Therefore, ts-WTA based disparity selective cell is an innovative design that could be used to create disparity selective maps in silicon with potential application in artificial vision systems which would learn from their environment as they operate.

## 6. Discussion

The paper discusses a novel application of the time-staggered Winner Take All algorithm and circuit to design a disparity selective neuron that learns different disparities through an adaptive learning mechanism based on the biological phenomenon of synapse elimination implemented using floating gate pMOS dynamics. It is well established that the brain is designed in a hierarchical fashion, where the lower cortical layers extract basic features from the input space and the higher layers use these basic features to extract more meaningful information. For example, in the visual cortex, the lower cortical layers extract basic features like left or right eye connectivity, orientation selectivity, direction selectivity, color, texture, and so forth. The cortical layers beyond these use these features to detect edges, depth, and so forth. A similar hierarchy of cortical processing is present in all sensory modalities.

The ts-WTA has been successfully used to form ocular dominance and orientation selective feature maps at the first layer of cortex. In this paper, we move one step up in the hierarchy by using the ts-WTA to extract information from ocular dominance patterns (layer 1) in the form of interlaced images from the left and the right eye as inputs going into a higher layer (layer 2), where disparity is detected. By exploiting the idea behind cortical hierarchy and competitive learning using ts-WTA, similar hierarchical feature maps can be created in other sensory modalities. Eventual integration of all these hierarchically organized adaptive feature maps, processing different sensory inputs, would lead to the formation of a generic and adaptive cortical structure that would to some extent capture the true essence of cortical plasticity and would lead to a new era of intelligent machines that would not rely on preprogramming or prewired hardware but would learn from experience just like the human brain does.

## Supplementary Material

A rigorous stress analysis under parameter variation has been performed on the designed disparity selective cell. The analysis brings out its robustness to parameter variation and its suitability to hardware implementation.

## Figures and Tables

**Table 1:** 

Model proposed	Hardware type	Method	Adaptability	Bioinspiration	Disparity range	Resolution	Power consumption
Shi and Tsang, 2003 [18]	Mixed analog & digital (use Gabor filter chips, AER Protocol, and Xilinx CPLD	Binocular energy model	Nonadaptive	Emulates disparity tuned complex cells	3 disparities	Low level vision	—

Díaz et al., 2007 [13]	Digital (use Gabors, FPGA based SOC that can be used in embedded systems)	Modified phase based technique	Adaptive (in the sense that it can dynamically adjust number of disparities)	Takes multiple disparity (estimates and integrates the results to emulate computations by many neurons in parallel)	FPGA can be configured to have flexible disparities depending on image, max (−24 to 24)	Subpixel resolution	—

Shimonomura et al., 2008 [19]	Mixed analog & digital (use aVLSI silicon retinas, Gabor chips to represent simple cells, & FPGA to compute disparity)	Energy model	Nonadaptive	Inspired by the hierarchical organization of simple and complex cell	5 disparities	Low level vision	225 mW

Mandal et al., 2010 [20]	Mixed analog & digital (use massively parallel SIMD Current-Mode Analog Matrix Processor and FPGA based microcontroller)	Binocular energy model	Nonadaptive	Bioinspired because it uses binocular energy model	3 disparities	Low level vision	250 mW

Rogister et al., 2012 [21]	Digital & software (use AER silicon retina for input and the rest of processing is done in software)	—	Nonadaptive	Inspired by the asynchronous event based dynamics of the brain	—	Low level vision	—

Our model	Pure analog, based on floating gate MOSFETs (uses ts-WTA as building block)	Position shift	Adaptive (the cell can learn any disparity during learning phase)	Takes inspiration from local, hierarchical processing, cortical plasticity, and columnar architecture of the brain	3 disparities but extendable to more disparities	Low level vision	180 mW (during learning phase) 60 mW (during detection phase)

## Appendix

A rigorous stress analysis under parameter variation has been done for the disparity selective cell designed and can be found in the Supplementary Material available online at http://dx.doi.org/10.1155/2016/8751874.

## References

[1] Lazaros N., Sirakoulis G. C., Gasteratos A. (2008). Review of stereo vision algorithms: from software to hardware. *International Journal of Optomechatronics*.

[2] Veksler O. Extracting dense features for visual correspondence with graph cuts.

[3] Gong M., Yang Y.-H. (2005). Fast unambiguous stereo matching using reliability-based dynamic programming. *IEEE Transactions on Pattern Analysis and Machine Intelligence*.

[4] Yoon K.-J., Kweon I. S. (2006). Adaptive support-weight approach for correspondence search. *IEEE Transactions on Pattern Analysis and Machine Intelligence*.

[5] Ohzawa I., DeAngelis G. C., Freeman R. D. (1990). Stereoscopic depth discrimination in the visual cortex: neurons ideally suited as disparity detectors. *Science*.

[6] Ohzawa I., DeAngelis G. C., Freeman R. D. (1996). Encoding of binocular disparity by simple cells in the cat's visual cortex. *Journal of Neurophysiology*.

[7] Sanger T. D. (1988). Stereo disparity computation using Gabor filters. *Biological Cybernetics*.

[8] Alonso J. D. (2006). *Multimodal bio-inspired vision system. High performance motion and stereo processing architecture [Ph.D. dissertation]*.

[9] Brown M. Z., Burschka D., Hager G. D. (2003). Advances in computational stereo. *IEEE Transactions on Pattern Analysis and Machine Intelligence*.

[10] Porr B., Nürenberg B., Wörgötter F. (2002). A VLSI-compatible computer vision algorithm for stereoscopic depth analysis in real-time. *International Journal of Computer Vision*.

[11] Hariyama M., Takeuchi T., Kameyama M. VLSI processor for reliable stereo matching based on adaptive window-size selection.

[12] Akin A., Baz I., Atakan B., Boybat I., Schmid A., Leblebici Y. A hardware-oriented dynamically adaptive disparity estimation algorithm and its real-time hardware.

[13] Díaz J., Ros E., Sabatini S. P., Solari F., Mota S. (2007). A phase-based stereo vision system-on-a-chip. *BioSystems*.

[14] Indiveri G., Horiuchi T. K. (2011). Frontiers in neuromorphic engineering. *Frontiers in Neuroscience*.

[15] Gupta P., Markan C. M. (2014). An adaptable neuromorphic model of orientation selectivity based on floating gate dynamics. *Frontiers in Neuroscience*.

[16] Mahowald M. Analog VLSI chip for stereocorrespondence.

[17] Erten G., Goodman R. M. (1996). Analog VLSI implementation for stereo correspondence between 2-D images. *IEEE Transactions on Neural Networks*.

[18] Shi B. E., Tsang E. K. (2003). A neuromorphic multi-chip model of a disparity selective complex cell. *Advances in Neural Information Processing Systems*.

[19] Shimonomura K., Kushima T., Yagi T. (2008). Binocular robot vision emulating disparity computation in the primary visual cortex. *Neural Networks*.

[20] Mandal S., Shi B., Dudek P. Binocular disparity calculation on a massively-parallel analog vision processor.

[21] Rogister P., Benosman R., Ieng S.-H., Lichtsteiner P., Delbruck T. (2012). Asynchronous event-based binocular stereo matching. *IEEE Transactions on Neural Networks and Learning Systems*.

[22] Blake R., Hirsch H. V. B. (1975). Deficits in binocular depth perception in cats after alternating monocular deprivation. *Science*.

[23] Yeshurun Y., Schwartz E. L. (1989). Cepstral filtering on a columnar image architecture: a fast algorithm for binocular stereo segmentation. *IEEE Transactions on Pattern Analysis and Machine Intelligence*.

[24] O'Dell C., Boothe R. G. (1997). The development of stereoacuity in infant rhesus monkeys. *Vision Research*.

[25] Birch E. E., Gwiazda J., Held R. (1982). Stereoacuity development for crossed and uncrossed disparities in human infants. *Vision Research*.

[26] Boothe R. G., Dobson V., Teller D. Y. (1985). Postnatal development of vision in human and nonhuman primates. *Annual Review of Neuroscience*.

[27] Held R. (1993). Two stages in the development of binocular vision and eye alignment. *Early Visual Development, Normal and Abnormal*.

[28] Franz A., Trieseh J. Emergence of disparity tuning during the development of vergence eye movements.

[29] Wiemer J., Burwick T., von Seelen W. (2000). Self-organizing maps for visual feature representation based on natural binocular stimuli. *Biological Cybernetics*.

[30] Lippert J., Fleet D. J., Wagner H. (2000). Disparity tuning as simulated by a neural net. *Biological Cybernetics*.

[31] Solgi M., Weng J. (2009). Developmental stereo: emergence of disparity preference in models of the visual cortex. *IEEE Transactions on Autonomous Mental Development*.

[32] Markan C. M., Gupta P., Bansal M. (2013). An adaptive neuromorphic model of ocular dominance map using floating gate ‘synapse’. *Neural Networks*.

[33] Kruger W. F., Hasler P., Minch B. A., Koch C. An adaptive WTA using floating gate technology.

[34] Chakrabartty S., Cauwenberghs G. (2007). Sub-microwatt analog VLSI trainable pattern classifier. *IEEE Journal of Solid-State Circuits*.

[35] Stent G. S. (1973). A physiological mechanism for Hebb's postulate of learning. *Proceedings of the National Academy of Sciences of the United States of America*.

[36] Draft R. W., Lichtman J. W. (2009). It's lonely at the top: winning climbing fibers ascend dendrites solo. *Neuron*.

[37] Misgeld T. (2011). Lost in elimination: mechanisms of axonal loss. *e-Neuroforum*.

[38] Turney S. G., Lichtman J. W. (2012). Reversing the outcome of synapse elimination at developing neuromuscular junctions in vivo: evidence for synaptic competition and its mechanism. *PLoS Biology*.

[39] Carrillo J., Nishiyama N., Nishiyama H. (2013). Dendritic translocation establishes the winner in cerebellar climbing fiber synapse elimination. *The Journal of Neuroscience*.

[40] Favero M., Busetto G., Cangiano A. (2012). Spike timing plays a key role in synapse elimination at the neuromuscular junction. *Proceedings of the National Academy of Sciences of the United States of America*.

[41] Sur M., Leamey C. A. (2001). Development and plasticity of cortical areas and networks. *Nature Reviews Neuroscience*.

[42] Wyatt R. M., Balice-Gordon R. J. (2003). Activity-dependent elimination of neuromuscular synapses. *Journal of Neurocytology*.

[43] Indiveri G. (2001). A current-mode hysteretic winner-take-all network, with excitatory and inhibitory coupling. *Analog Integrated Circuits and Signal Processing*.

[44] Mead C. A., Lazzaro J., Ryckebusch S., Mahowald M. A. (1988). Winner-take-all networks of *O*(*N*) complexity. *Advances in Neural Information Processing Systems*.

[45] Chen G., Lu H. D., Roe A. W. (2008). A map for horizontal disparity in monkey V2. *Neuron*.

[46] Ferster D. (1981). A comparison of binocular depth mechanisms in areas 17 and 18 of the cat visual cortex. *The Journal of Physiology*.

[47] Julesz B. (1960). Binocular depth perception of computer-generated patterns. *Bell System Technical Journal*.

[48] Julesz B. (1971). *Foundations of Cyclopean Perception*.

[49] Poggio G. F., Fischer B. (1977). Binocular interaction and depth sensitivity in striate and prestriate cortex of behaving rhesus monkey. *Journal of Neurophysiology*.

[50] Poggio G. F. (1995). Mechanisms of stereopsis in monkey visual cortex. *Cerebral Cortex*.

[51] Hubel D. H., Wiesel T. N. (1970). Stereoscopic vision in macaque monkey: cells sensitive to binocular depth in area 18 of the macaque monkey cortex. *Nature*.

[52] Maunsell J. H. R., Van Essen D. C. (1983). Functional properties of neurons in middle temporal visual area of the macaque monkey. I. Selectivity for stimulus direction, speed, and orientation. *Journal of Neurophysiology*.

[53] Hubel D. H., Livingstone M. S. (1987). Segregation of form, color, and stereopsis in primate area 18. *Journal of Neuroscience*.

[54] Vanetti M., Gallo I., Binaghi E. Dense two-frame stereo correspondence by self-organizing neural network.

[55] Chklovskii D. B., Koulakov A. A. (2004). Maps in the brain: what can we learn from them?. *Annual Review of Neuroscience*.

[56] Erwin E., Obermayer K., Schulten K. (1995). Models of orientation and ocular dominance columns in the visual cortex: a critical comparison. *Neural Computation*.

