# Supplementary material for: Adaptive Neuromorphic Circuit for Stereoscopic Disparity Using Ocular Dominance Map
Source: Neurosci J. 2016 May 3;2016:8751874. doi: 10.1155/2016/8751874 (PMC4868909; doi:10.1155/2016/8751874)
Supplement: Supplementary file 1 — A rigorous stress analysis under parameter variation has been performed on the designed disparity selective cell. The analysis brings out its robustness to parameter variation and its suitability to hardware implementation. [file 8751874.f1.pdf]

1. Noise Analysis

To test the circuits for robustness against noise, noise analysis was performed both on the individual ts-WTA cell and the Disparity Selective Cell. In the design of the ts-WTA cell, the common source node from which the tunnel and injection feedback (that modifies the floating gate voltages) originates is the most sensitive node. Therefore, the resistance to noise at this node is critical for circuit performance. Noise was applied at this node and both the frequency and amplitude of noise was varied from 0.01s to .1s and ± 0.1 mV to 100mV respectively. It was observed that while there wasn't much effect of the frequency of noise, the amplitude of noise started affecting the circuit performance beyond ± 90mV. Beyond this it was unable to distinguish between the two inputs coming to the two synapses. For the Disparity Selective Cell, noise was applied to the common source node of each of the 9x9 ts-WTA cells. For the disparity cell also, the noise frequency did not have any effect however, the circuit's performance or its ability to learn a particular disparity pattern degraded beyond ± 90mV which is fairly high. We can say that both the circuits are fairly robust to noise.

2. Temperature Analysis

To test the robustness of the ts-WTA cell and the Disparity Selective Cell under temperature variation, simulations were performed for different temperatures in the range -45 to 85 ^{o}C. It was found that the ts-WTA worked well between -45 ^{o}C and 65 ^{o}C, however with an increase in temperature there was an increase in the learning time. This delayed learning time in the ts-WTA at high temperatures seemed to be affecting the Disparity Cells performance. It appears that the increased learning time increases the diffusion or the neighborhood influence on each cell, as a result of which the pattern that the disparity cell learns is a not a unique pattern but a reflection of many input patterns. This can be adjusted by changing the diffusion resistances, however this aspect has not been taken into account in the current circuit.

During the detection phase, the circuit's performance remains unaltered for low temperatures. However for higher temperatures, although the detection of disparity happens correctly, there is a reduction in the output voltage range.

3. Monte Carlo Analysis of Disparity Selective Cell

To test the robustness of our Disparity Selective Cell, Monte Carlo Analysis with random parameter value variations was performed. The Disparity Selective Cell's performance heavily depends on the injection and tunnel currents. Any variation in these currents can affect the equilibrium of the circuit and affect the circuit's learning and response behavior. Our models for injection and tunnel currents are based on the equations described in [Rahimi et.al, 2001] also described below.

The tunnel current varies according to the below equation and depends on the floating gate voltage, the tunnel voltage and a factor V_f that depends on the oxide thickness. Our model assumes an oxide thickness of 70A^{o}. I_{to} is a pre-exponential current. The typical values of these parameters are listed in table I.

$$I_{tunnel} = F_{Tun}(V_{Tun}, V_{fg}) = I_{to} \times W \times L \times \exp\left(-\frac{V_f}{V_{Tun} - V_{fg}}\right) \quad (1)$$

The injection current varies according to the below equation. It depends on the gate (floating)to drain and source to drain voltages. Here η, β and δ are fit parameters and λ =1 for units consistency. I_S is the source current which is ~10nA and can be ignored for all practical purposes. The typical values of the parameters η, β and δ are listed in table I.

$$I_{injection} = F_{Inj}(V_d, I_s, V_{fg}) = \eta \times I_s \times \exp\left(-\frac{\beta}{(V_{fg} - V_d + \delta)^2} + [\lambda \times (V_s - V_d)]\right) \quad (2)$$

Table I. Base values of all the device parameters

**Parameter**	**I_{to} (A/m²)**	**V_f (V)**	**η**	**β**	**δ**
**Base Value**	9.35×10^8	368.04	1.30×10^{-5}	155.75	0.702

A single Disparity Cell is made from 9x9 ts-WTA cells. During fabrication, the variation in parameters can happen in two ways.

i). There could be variation in the parameter base values over the whole IC

ii). There could be minor variations in parameter values across the same IC

To test the robustness of our design under these two situations we performed Monte-Carlo analysis at two levels. First, by randomly varying the base values of all the parameters and applying the same (randomly generated parameters) to all the 9x9 ts-WTA cells and second, by randomly varying the base values and applying different parameter values to all 9x9 ts-WTA cells. The first analysis determines the extent to which the disparity cell is resilient to changes in the base values of parameters and the second analysis checks for how resilient the circuit is to variations in parameters across the 9x9 ts-WTA cells over the same IC. The following sections describe the detailed analysis.

3.1 Performance under parameter base value variation

To check for the first case, a MATLAB code was written to generate random values of all the parameters. The parameters were varied by 10%, 5% and 3% from the base values listed in table I. Some sample values of the parameters are listed in tables 2a, 2c and 2e.

3.1.1 Performance under 10% parameter variation

Multiple simulations were performed on the Disparity cell keeping the inputs and initial conditions the same but varying the parameters and applying the same to all the 9x9 ts-WTA cells.

Table 2a. 10% variation in device parameters

	I_{to} (A/m^2)	V_f (V)	η	β	δ
1	987114888.228762	362.893919	0.000013	145.053742	0.677823
2	875529355.768128	364.844702	0.000014	140.724017	0.663066
3	***925538930.197264***	***343.556415***	***0.000012***	***166.233249***	***0.736087***
4	***947332216.259480***	***396.207331***	***0.000014***	***144.205576***	***0.730049***
5	***1016157953.038652***	***385.248023***	***0.000013***	***161.530224***	***0.663095***
6	***844396973.116741***	***358.564317***	***0.000014***	***169.719006***	***0.699921***
7	***849825326.820521***	***350.038661***	***0.000012***	***159.342702***	***0.764429***
8	882827929.914150	362.929978	0.000014	148.026625	0.680760
9	***966722055.874403***	***374.177837***	***0.000014***	***145.640335***	***0.747585***
10	***856079594.191899***	***358.490477***	***0.000012***	***163.461846***	***0.692258***

Analysis: The cell's learning response remained fairly stable for cases 1, 2 and 8 however for rest of the cases the response was significantly altered. Prior analysis done on a single ts-WTA cell reported in [Markan, C.M., Gupta, P., Bansal, M., 2013] shows that ts-WTA is stable under 10% variations in all parameters except the value of parameter V_f. However, as can be seen from equation(1), even when V_f changes, the overall effect of the exponential term in the tunnel current can be kept constant by changing the tunnel voltage(V_{tun}) appropriately. By modifying V_{tun}, for all the cases except for case 4, the response of the cell could be made normal.Therefore, it seems that the circuit is not very stable to 10% variation in V_f. However, in 90% of the cases, we can recover from this unstable response by adjusting V_{tun}. The exact variation in V_{tun} can be seen in table 2b.

Table 2b. Change in the value of $V_{tun}$from the original value of 13.6v

Case No	***3***	***5***	***6***	***7***	***9***	***10***
Modified V_{tun}(volts)	13	14	13.3	13.3	13.8	13.3
δV_{tun}(volts)	-0.6	0.4	-0.3	-0.3	0.2	-0.3

3.1.2 Performance under 5% parameter variation

To check the if the performance of the disparity cell improves with a lower percentage parameter variation, we varied the parameters by 5%. Some of the sample parameter values used in the simulations are recorded in table 2c.

Table 2c. 5% variation in device parameters

	I_{to} (A/m²)	V_f (V)	η	β	δ
1	***903448852.215753***	***351.649108***	***0.000013***	***155.776379***	***0.734687***
2	***945800047.304039***	***380.988332***	***0.000013***	***160.131355***	***0.696717***
3	***888654705.346983***	***377.145651***	***0.000013***	***158.178012***	***0.706970***
4	953635366.477950	360.011766	0.000013	158.136659	0.733318
5	911184212.488790	368.653780	0.000013	148.067916	0.719875
6	924005782.434137	358.665951	0.000013	154.696435	0.708818
7	912696144.559396	360.404296	0.000013	148.440872	0.681787
8	***945049412.311427***	***383.577475***	***0.000013***	***149.460724***	***0.718531***
9	945380010.191412	362.462831	0.000013	152.474247	0.711202
10	914740138.349817	375.717765	0.000013	159.974569	0.683564

Analysis: The cells response remained fairly stable for 60% of the cases, however, in 40% cases (e.g. case 1, 2, 3 and 8) the learning was altered moderately. In these cases also the response could be corrected by modifying V_{tun}appropriately. The exact change in $V_{tun}$is listed in table 2d.

Table 2d. Change in the value of V_{tun} from the original value of 13.6v

Case No	***1***	***2***	***3***	***8***
Modified V_{tun}(volts)	13.3	13.8	13.8	14
δV_{tun}(volts)	-0.3	0.2	0.2	0.6

3.1.3 Performance under 3% parameter variation

To find the range of parameter variation within which the cell works perfectly (without having to change V_{tun}) we then varied the parameters by 3%. Some sample values are listed in table 2e.

Table 2e. 3% variation in device parameters

	I_{to} (A/m²)	V_f (V)	η	β	δ
1	942305113.731676	357.863394	0.000013	155.912888	0.700073
2	919794968.390765	361.952084	0.000013	154.635215	0.704436
3	956106758.514632	376.302343	0.000013	151.379141	0.702056
4	917102887.966741	372.542313	0.000013	151.709425	0.702733
5	912307660.991632	359.245728	0.000013	154.072777	0.681407
7	938824854.542024	365.572080	0.000013	155.638304	0.706797
8	947710289.185848	365.296566	0.000013	157.926790	0.700346
9	933122974.181908	360.571770	0.000013	153.492817	0.721009
10	943420647.202232	362.339327	0.000013	155.784875	0.690321

Analysis: It was found that in all the cases the cell's behavior was as expected. For case 3, the learning took slightly longer, however the output or receptive field did converge to the expected pattern. For all other cases the learning was normal.

3.2 Performance under parameter variation across the same IC

To test how robust our circuit is to parameter variations between the different 9x9 ts-WTA cells a MATLAB code was written to generate random parameters for all the 81 ts-WTAs. The parameter variation range was varied from $\pm 2\%$ to $\pm 10\%$. It was found that when the parameters varied within $\pm 3\%$ of the base values, the cell performed normally, however, for larger limits the cell's performance deteriorated. Sample values with a $\pm 3\%$ random parameter variation across all 9x9 ts-WTA cells are listed in table 3.

Table 3. Sample values of Monte-Carlo analysis with $\pm 3\%$ parameter variation over all 81 ts-WTA cells together

tsWTA	I_{to} (A/m^2)	V_f (V)	η	β	Δ
1	916926258.638770	374.388765	0.000013	154.220189	0.686514
2	938515684.395454	373.868665	0.000013	158.703084	0.707317
3	943198321.797023	371.029368	0.000013	153.853756	0.685235
4	936013995.315444	363.245351	0.000013	152.389534	0.710212
5	943216764.482654	360.545627	0.000013	151.369412	0.681143
6	934960249.771276	370.054539	0.000013	152.911303	0.685080
7	942163018.938486	367.354815	0.000013	151.891182	0.698130
8	934978692.456907	359.570799	0.000013	159.771959	0.680987
9	939661329.858476	378.813901	0.000013	154.922632	0.701025
10	961250755.615160	378.293801	0.000013	159.405526	0.721829
11	909833393.016729	375.454503	0.000013	154.556199	0.699747
12	958749066.535150	367.670486	0.000013	153.091976	0.682604
13	909851835.702361	364.970763	0.000013	152.071855	0.695654
14	957695320.990982	374.479675	0.000013	153.613746	0.699592
15	908798090.158193	371.779951	0.000013	152.593624	0.712642
16	957713763.676613	363.995934	0.000013	151.129402	0.695499
17	962396401.078182	361.156637	0.000013	155.625075	0.715537
18	927885826.834867	360.636536	0.000013	160.107969	0.694220
19	932568464.236436	357.797239	0.000013	155.258642	0.714258
20	925384137.754857	372.095622	0.000013	153.794419	0.697116
21	932586906.922067	369.395898	0.000013	152.774298	0.710166
22	924330392.210689	378.904810	0.000013	154.316189	0.714103
23	931533161.377899	376.205087	0.000013	153.296067	0.685033
24	924348834.896320	368.421070	0.000013	151.831845	0.710011
25	929031472.297889	365.581773	0.000013	156.327517	0.687929
26	950620898.054573	365.061672	0.000013	151.465412	0.708732
27	955303535.456142	362.222375	0.000013	155.961084	0.686650
28	948119208.974563	376.520758	0.000013	154.496862	0.711627
29	913608634.731247	376.000657	0.000013	158.979756	0.690310
30	947065463.430395	361.247546	0.000013	155.018631	0.686495
31	939881136.948816	375.545929	0.000013	153.554409	0.711472
32	947083906.116026	372.846206	0.000013	152.534287	0.682402
33	910053200.107069	372.186531	0.000013	153.187976	0.710193
34	917255969.274280	369.486808	0.000013	152.167854	0.681124
35	907551511.027059	361.563217	0.000013	156.219426	0.713089
36	914754280.194270	358.863493	0.000013	155.199304	0.684019
37	936343705.950954	358.343393	0.000013	159.682199	0.704822
38	952147850.323374	375.212011	0.000013	156.885460	0.707324
39	945094128.509911	370.251336	0.000013	157.778615	0.721689
40	946588241.534838	361.889786	0.000013	151.110136	0.710229

**41**	948082793.619080	378.515306	0.000013	158.072742	0.721210
**42**	926641976.156827	368.470338	0.000013	158.521796	0.705382
**43**	956910280.479333	370.277375	0.000013	152.741519	0.712188
**44**	921082367.368290	377.230513	0.000013	152.746472	0.708287
**45**	951350671.690796	379.037550	0.000013	156.311195	0.715094
**46**	938458128.126249	368.496377	0.000013	153.484701	0.695881
**47**	931404406.312785	363.535702	0.000013	154.377856	0.710246
**48**	932898519.337712	377.256552	0.000013	157.054377	0.698786
**49**	961718880.124376	361.631085	0.000013	153.783781	0.691501
**50**	912952253.959702	361.754704	0.000013	155.121037	0.693939
**51**	914446366.984628	375.475554	0.000013	157.797558	0.682479
**52**	907392645.171165	370.514879	0.000013	158.690713	0.696844
**53**	908886758.196092	362.153329	0.000013	152.022234	0.685385
**54**	952094214.631544	373.694556	0.000013	158.540739	0.708292
**55**	945040492.818081	368.733881	0.000013	159.433894	0.722657
**56**	946534605.843008	360.372331	0.000013	152.765415	0.711198
**57**	919254966.629671	366.829264	0.000013	158.839819	0.703912
**58**	926588340.464997	366.952883	0.000013	160.177075	0.706350
**59**	928082453.489924	358.591333	0.000013	153.508596	0.694890
**60**	921028731.676461	375.713058	0.000013	154.401751	0.709255
**61**	922522844.701387	367.351508	0.000013	157.078272	0.697796
**62**	951343205.488051	373.808442	0.000013	153.807676	0.690510
**63**	958676579.323377	373.932060	0.000013	155.144933	0.692948
**64**	960170692.348304	365.570510	0.000013	157.821454	0.681489
**65**	932891053.134967	372.027444	0.000013	154.550858	0.716323
**66**	940224426.970293	372.151062	0.000013	155.888114	0.718761
**67**	941718539.995220	363.789512	0.000013	158.564635	0.707301
**68**	934664818.181756	358.828837	0.000013	159.457790	0.721667
**69**	936158931.206683	372.549687	0.000013	152.789311	0.710207
**70**	908879291.993347	379.006621	0.000013	158.863715	0.702921
**71**	916212665.828673	357.047839	0.000013	160.200971	0.705359
**72**	917706778.853599	370.768689	0.000013	153.532492	0.693900
**73**	919201330.937842	365.311809	0.000013	151.150098	0.704880
**74**	912147170.065063	357.446464	0.000013	157.102168	0.696805
**75**	928028817.798094	357.073877	0.000013	155.163876	0.695859
**76**	948300904.687052	364.027016	0.000013	155.168828	0.691958
**77**	922469209.009558	365.834052	0.000013	158.733552	0.698764
**78**	922320701.160361	377.063633	0.000013	156.628128	0.715260
**79**	942484766.291424	376.017635	0.000013	159.875657	0.702075
**80**	929585636.105684	366.066372	0.000013	156.706191	0.689867
**81**	938236223.258724	357.886154	0.000013	158.417267	0.705530

5. Conclusions

i) The cell is fairly robust to noise

ii) Works well between -45 ^{o}C and 65 ^{o}C

iii) The two stage Monte-Carlo analysis performed on the Disparity Selective Cell brings forth the following conclusions

   a. The cell is fairly stable under a $\pm 3\%$ variation in parameter base values.

b. For a variation greater than 3% but less than 10%, the response of the cell gets altered but it can easily be recovered by changing the tunnel voltage V_{tun} appropriately.
c. The cell is also resilient upto a $\pm 3\%$ parameter mismatch between the 9x9 ts-WTA cells forming the disparity cell.

4. References

[1]. Rahimi, K., Diorio, C., Hernandez, C., &Brockhausen, M. D. (2002). A simulation model for floating-gate MOS synapse transistors. In *Circuits and Systems, 2002. ISCAS 2002. IEEE International Symposium on* (Vol. 2, pp. II-532). IEEE.
[2]. Markan, C. M., Gupta, P., & Bansal, M. (2013). An adaptive neuromorphic model of Ocular Dominance map using floating gate 'synapse'. *Neural Networks*.
